# 

**Published:** 1885-10

**Authors:** 


### OUR ADVERTISERS.

SEE advertisement of W. H. Smith & Co., page 38.

M. J. BREITENBACH, NEW YORK.—See advertisement of this gentleman, to be found on page 12, and write for samples of his preparation. They are highly recommended.

LIQUID BREAD.—This is the name given to a preparation of pure malt extract, by David Nicholson, St. Louis. It is the most pleasant preparation of malt we know, and is rapidly growing into popular favor. See the advertisement, page 35.

PAPINE.—Dr. H. M. Simmons, of Oak Hill, N. Y., says : I have been using Papine for the past three months, and must say it acts admirably, relieving pain and producing sleep without the unpleasant effects which we so often get from Morphine.

FELLOWS' SYRUP HYPOPHOSPHITES.—The reputation of this preparation is too well known to require any words of commendation from us. To those of our readers who have not given it a thorough trial, we would say send for a sample bottle at once and give it a trial. You will be pleased with it.

SURGICAL INSTRUMENTS.—The instrument house of Isaac Phillips, at 13 N. Broad street, Atlanta, is the only house in the South dealing exclusively in surgical instruments and appliances. He has a very full line of dissecting cases, to which he invites the attention of medical students.

MAGNUS & HIGHTOWER, ATLANTA.—Attention is directed to the advertisement of this house, to be found on page 6. In addition to their large stock of drugs, chemicals, etc., they carry a full line of surgical instruments. They invite special attention of medical students to their large stock of dissecting cases.

BUFFALO LITHIA WATER.—Dr. Hunter McGuire, the well-known surgeon of Richmond, says : "Buffalo Lithia Water, Spring No. 2, as an *Alkaline Diuretic*, is invaluable. In *Uric Acid Gravel*, and indeed in diseases generally dependent upon a *Uric Acid Diathesis*, it is a remedy of extraordinary potency. I have prescribed it in cases of *Rheumatic Gout*, which had resisted the ordinary remedies, with wonderfully good results. I have used it also in my own case, being a great sufferer from this malady, *and have derived more benefit from it than from any other remedy*. It has very marked adaptation in diseases of the *Digestive Organs*. In that condition, especially known as *Nervous Dyspepsia*, frequently caused by over-mental labor, and in those cases also where there is *excess of Acid* in the process of nutrition, it will be found highly efficacious.

